# Building a semantically annotated corpus for chronic disease complications using two document types

**DOI:** 10.1371/journal.pone.0247319

**Published:** 2021-03-18

**Authors:** Noha Alnazzawi

**Affiliations:** Department of Computer Science and Engineering, Royal Commission for Jubail and Yanbu, Yanbu University College, Yanbu Industrial City, Saudi Arabia; Doctrine, FRANCE

## Abstract

Narrative information in electronic health records (EHRs) contains a wealth of information related to patient health conditions. In addition, people use Twitter to express their experiences regarding personal health issues, such as medical complaints, symptoms, treatments, lifestyle, and other factors. Both genres of text include different types of health-related information concerning disease complications and risk factors. Knowing detailed information about controlling disease risk factors has a great impact on modifying these risks and subsequently preventing disease complications. Text-mining tools provide efficient solutions to extract and integrate vital information related to disease complications hidden in the large volume of the narrative text. However, the development of text-mining tools depends on the availability of an annotated corpus. In response, we have developed the PrevComp corpus, which is annotated with information relevant to the identification of disease complications, underlying risk factors, and prevention measures, in the context of the interaction between hypertension and diabetes. The corpus is unique and novel in terms of the very specific topic in the biomedical domain and as an integration of information from both EHRs and tweets collected from Twitter. The annotation scheme was designed with guidance by a domain expert, and two further domain experts performed the annotation, resulting in a high-quality annotation, with agreement rate F-scores as high as 0.60 and 0.75 for EHRs and tweets, respectively.

## I. Introduction

Chronic diseases, including stroke, cancer, hypertension, diabetes and chronic respiratory conditions, constitute the leading cause of death in the world, and their impact is steadily growing.

Hypertension and diabetes are common comorbidities. There is a vicious cycle between the two diseases, as hypertension occurs twice as frequently in patients with diabetes compared with those who do not have diabetes. Moreover, patients with hypertension often exhibit insulin resistance and are at greater risk of developing diabetes than normotensive individuals [1]. Over time, diabetes and hypertension conditions can become worse and cause many complications that can affect any part of the patient’s body.

Both hypertension and diabetes share the same complications; these complications can be divided into macrovascular and microvascular disorders [2]. Microvascular complications include retinopathy, nephropathy, and neuropathy; macrovascular complications include coronary artery disease (CAD), myocardial infarction, congestive heart failure, stroke, and peripheral vascular disease, resulting in organ and tissue damage in approximately one-third to one-half of people with diabetes [3, 4].

Risk factors are defined as any medical condition or social behavior that increases the likelihood of developing a disease, worsening an existing medical condition, or causing severe complications [5]. There is significant overlap in the risk factors for developing macrovascular and microvascular diseases for patients with hypertension and diabetes. Early interventions to control disease risk factors have the potential to modify these risks and subsequently prevent or reduce the chance of developing disease complications and hence improve global health and life expectancy [6].

Different textual sources, including EHRs and social media, provide a vital source of related information on chronic disease complications and the degree of their severity, such as risk factors and prevention measures. EHRs are written by clinicians at the point of care and provide greater detail about patient health conditions, such as symptoms, medical history, the results of clinical examinations, laboratory tests performed and their results, and prescribed medicines [7]. However, this important information about a patient’s health is hidden within the narrative text of EHRs. Therefore, it is often necessary for doctors to read through these narratives to gain a full picture of a patient’s history of a disease to determine how to control the condition, to stop it from becoming worse or to prevent any complications from occurring. However, it is very time consuming for clinicians to go through large volumes of text to find snippets of potentially useful information that are locked away in unstructured text format.

Online social media is producing massive amounts of information on an unprecedented scale, with 19%-28% of all users participating in health discussions [8]. On Twitter, over 500 million users worldwide discuss their life experiences, social factors, lifestyle, and health conditions [9]. Users often share a combination of their health conditions, related symptoms, social factors and their impact on their health conditions rather than providing a suspected or final diagnosis. The information that the users share on Twitter might not have been provided to their clinicians; thus, it is a potential source of new information to guide clinical decision making for the prevention or delay of disease onset [10]. Despite its importance, this topic has not yet been extensively studied [9].

While EHRs and Twitter include different health-based information related to disease complications that may complement each other well, both contain information on chronic disease that is locked away within large volumes of unstructured text, which creates a massive challenge for clinicians and researchers and for the development of computerized applications. Furthermore, the text of EHRs and Twitter have different characteristics and features. For example, the text in EHRs is handwritten by clinicians, is full of domain abbreviations and contains a high level of lexical and semantic variability. Texts uploaded to Twitter are written by nonclinical individuals and are limited to 280 characters per tweet. Therefore, users of Twitter are limited to using a small number of characters to express and share their health experiences, including the status of medical conditions, signs and symptoms, drug reactions, etc. Text mining (TM) tools provide efficient means to automate the process of extracting and integrating vital information from different text types on disease complications that affect patient health. To enhance the automatic extraction and integration of chronic disease complications from two textual sources that are known to reflect different aspects of chronic disease complications, the contribution of this article is twofold:

1. We have created a new corpus (PrevComp) that is annotated by domain experts with several types of information on disease complications related to interactions between hypertension and diabetes, including macrovascular and microvascular conditions, risk factors and prevention measures. To ensure that the tools developed using the corpus are robust for multiple text types, the corpus integrates text from heterogeneous sources, i.e., EHRs and text from social media (i.e., Twitter).
2. We enriched the annotations with a link to UMLS Metathesaurus concepts to facilitate research on ML-based normalization methods to automatically integrate information on disease complications obtained from EHRs and Twitter.

The corpus is freely available to stimulate the development of TM systems for the automatic extraction and integration of details relating to chronic disease complications from the free text of EHRs and tweets. The developed TM systems can ultimately be applied to support evidence-based healthcare and clinical decision support systems.

## II. Related work

The development of TM tools depends on the availability of an annotated corpus that serves as a resource to train and evaluate the TM tools. In the clinical domain, several corpora have been constructed to promote the development of TM tools to extract and integrate vital information hidden within the large volume of unstructured text. The developed corpora vary in terms of text types, annotation granularity and annotation approach. The annotation level can be divided into document level and fine-grained text-bound annotations that encode the exact locations (i.e., text span) of the annotated entity within the text [11, 12]. The corpus that is annotated at the document level is more suitable for developing and evaluating information retrieval methods rather than supporting the extraction of fine-grained information. On the other hand, corpora enriched with text-bound annotations provide detailed semantic and fine-grained annotations.

There are three approaches that can be used to annotate the clinical text:

1. Total manual annotation that starts from scratch and the text is entirely annotated by humans based on their knowledge.
2. Semiautomatic annotation in which the text is preannotated by an annotation tool and then the annotated text is reviewed by human experts to correct or add annotations.
3. Ontology-based annotation in which only terms present in the knowledge source will be annotated.

Each of the above approaches has advantages and disadvantages [13]. For example, ontology-based annotation suffers from limiting the annotation entities only to the terms provided in the ontology. Assisted annotation is more consistent and accelerates the process of manual annotations but may be biased (i.e., failure to annotate concepts completely missed by automatic annotation due to reliance on visual cues). Manual annotation of each document by more than one annotator can help to decrease the potential bias. However, manual annotation is very costly in terms of time and money.

Due to the difficulty of obtaining and sharing medical records, few annotated corpora have been made publicly available to the research community. Most of these corpora come in the form of shared tasks such as i2b2 [14], ShARe/CLEF [15, 16], and SemEval [17]. Recently, many research efforts have been made to develop a corpus of heterogeneous text sources to allow the development of robust TM systems that can extract and integrate relevant information from complementary text sources. For example, PhenoCHF [18] and COPD [19, 20] are collections of EHRs and research articles obtained from the literature. Both corpora have proven to be very useful and have been used to develop TM tools to extract and integrate phenotype information. Table 1 shows a summary of the characteristics of some of the well-known corpora in the clinical domain.

**Table 1:** Summary of the characteristics of some of the well-known corpora in the clinical domain.

Corpus	Document type	Semantic types	Annotation approach	Annotation level
I2b2 recognizing obesity and its comorbidities [21]	Discharge summaries	Obesity and its comorbidities	Manual	Document-level
I2b2 concept and relations [22]	Discharge summaries and progress notes	Problem, treat and test		Text-bound annotation
I2b2 identifying heart disease risk factors [14]	Longitudinal clinical narratives	CAD risk factors	Manual	Document-level
ShARe/CLEF [16]	Different clinical record types	Disorders and mapping the spans to UMLS concepts	Manual	Text-bound annotation
COPD [19]	1000 clinical records and 30 full-research papers	Problem, treatment, test	Semiautomatic	Text-bound annotation
PhenoCHF [18]	Clinical records and 10 full- research papers	CHF phenotypic information (causes, risk factors, signs and symptoms and nontraditional risk factors)	Manual	Text-bound annotation

In recent years, researchers have recognized that social media platforms can also provide important information related to public health [23–28]. Social media in general and Twitter in particular have been found to be useful and impactful resources in health-related studies [9].

Twitter-based health research is a growing field, as evidenced by the increasing number of publications per year and the diversity of funding organizations [23]. Several studies regarding the retrieval of health information from social media have already been published, with a major focus on content for sentiment analysis, image analysis [29] disease outbreaks [30–32], social behavior such as physical activity, smoking or alcohol use [33–35], pharmacovigilance adverse drug reactions [36], public health surveillance [37, 38], and predictions of disease prevalence [23, 39, 40].

The most commonly discussed disease-related topics on Twitter include important high morbidity and mortality conditions, such as influenza, cancer, and Ebola, and social behaviors, such as smoking and sleep issues. It is interesting that many of the most prevalent and costly chronic diseases, including diabetes and hypertension, have been less frequently investigated in previous studies. Despite the fact that the economic impact of hypertension and diabetes is an enormous burden on society, with estimated annual costs of $174 billion for diabetes care and $76.6 billion for hypertension-related problems [2, 3, 41], none of the previous studies focused specifically on chronic diseases, making it difficult to derive conclusions and recommendations in this specific and diverse domain.

## III. Methods

### A. Corpus construction

The PrevComp corpus consists of two document types: EHRs and tweets. The EHRs are a subset of the i2b2 heart risk factor EHR challenge [14]. The corpus consists of 1304 records annotated for CAD risk factors, including hypertension, hyperlipidemia, obesity, smoking status, family history, and diabetes. The corpus was annotated at the document level for the mentions of the risk factors or the indicators that suggest the presence of the medical conditions. After close consultation with a medical expert who is an internal medicine doctor and functioned as a guide and judge through the annotation process, the records were filtered, and only the records for patients known to have both hypertension and diabetes conditions were retained, resulting in 274 records. The tweets were collected from Twitter using the TweetScraper [42] method for the period between 01-01-2010 and 30-12-2019. The following list of keywords was used to collect the relevant tweets: hypertension, HTN, high blood pressure, diabetes, and diabetes mellitus. Those keywords were chosen by the medical experts who suggested the list and synonyms, resulting in 14,212 identified tweets that contained mentions of both target medical conditions. The tweets were further filtered by the annotators, and only tweets that included information directly related to our task in question (i.e., the interactions between hypertension and diabetes) were retained, resulting in 2,265 tweets that constitute the tweet subset of PrevComp.

**Fig 1:**
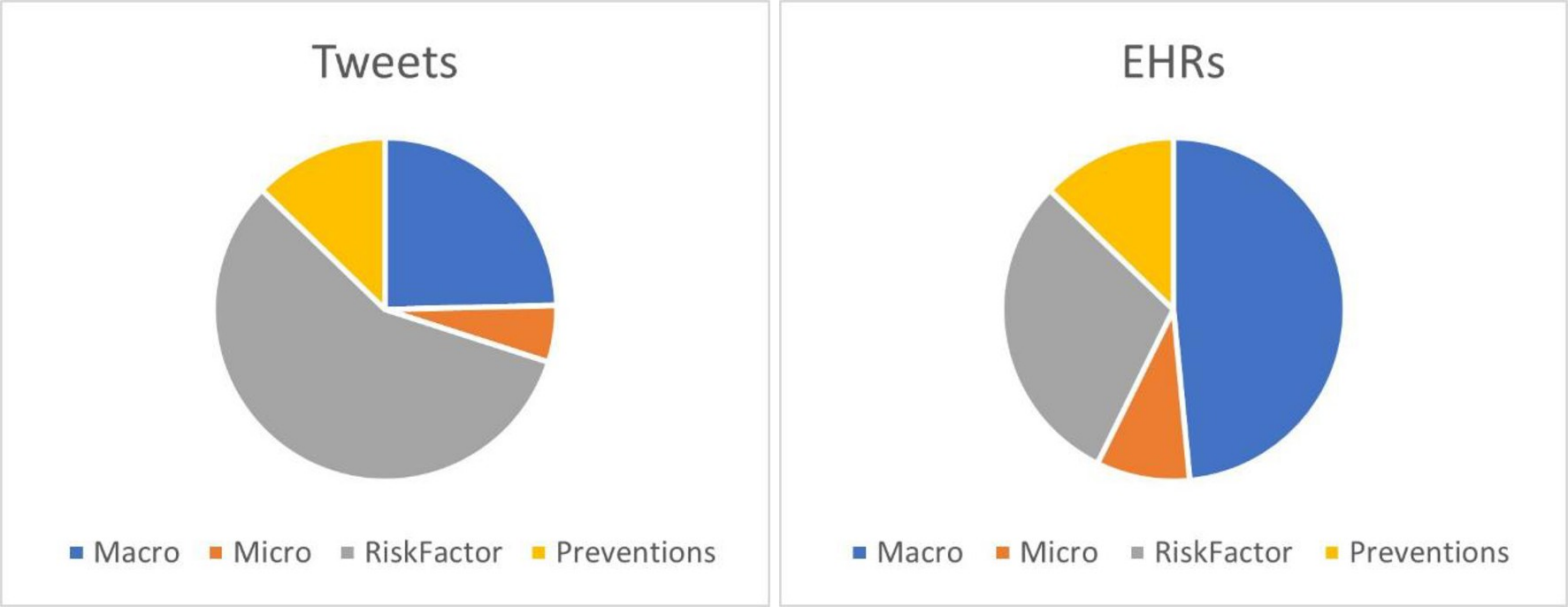
Distribution of the entity types in the PrevComp corpus.

Fig 1 describes the most common macrovascular and microvascular complications, risk factors and prevention strategies in the corpus and their distributions in the EHRs and tweets. In the EHRs, there was a large emphasis on describing the patient’s macrovascular complications resulting from having hypertension and diabetes conditions, but these played a much less significant role in tweets, where the dominant topics were risk factors that led to complications. In addition, it was noted that mentions of prevention strategies in the tweets were more common than their occurrence in EHRs.

### B. Annotation scheme and guidelines

**Fig 2:**
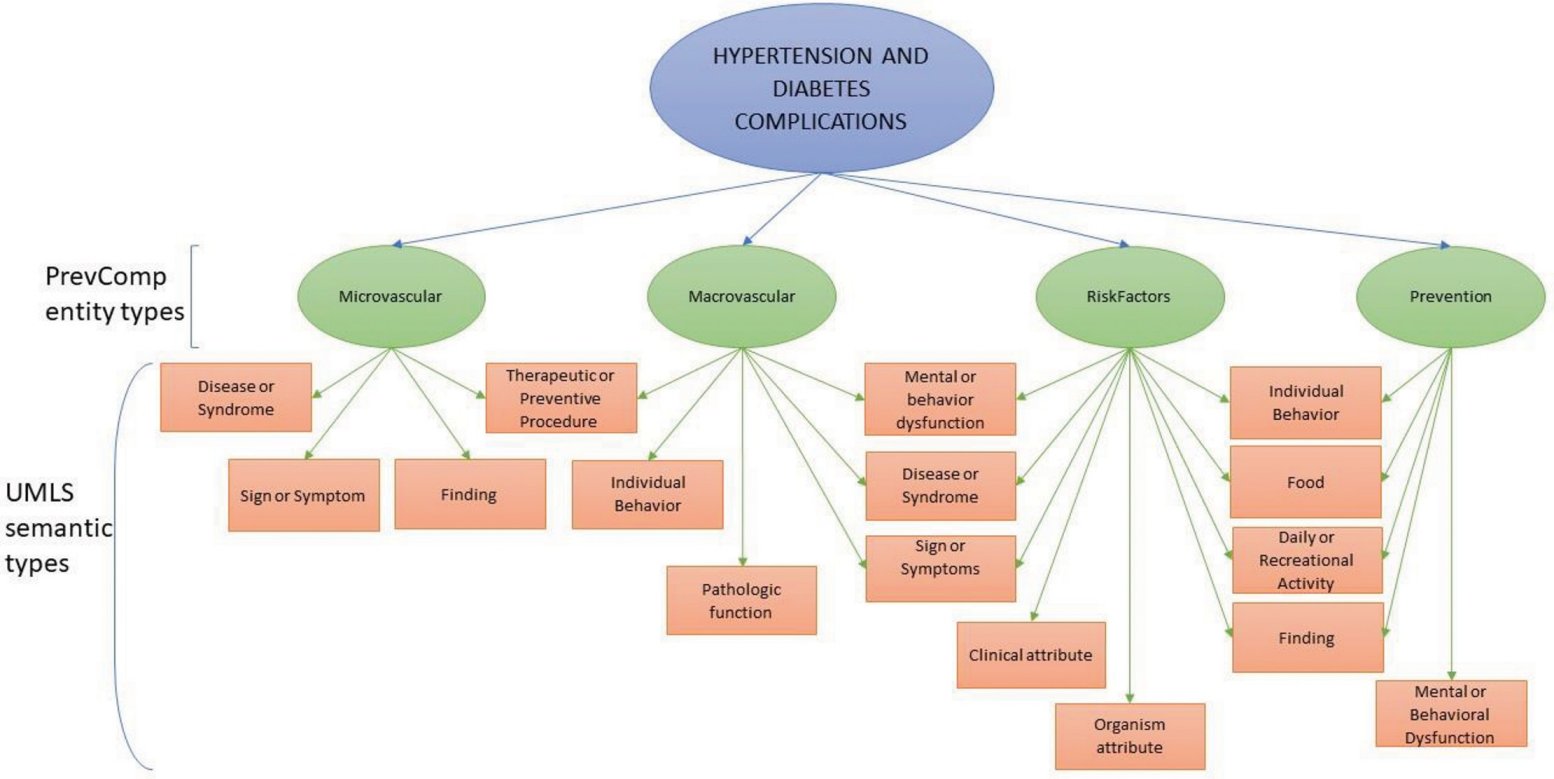
Annotation schema.

To ensure the relevance of the scheme to our research goals, we worked closely with a medical expert who is an internal medicine doctor and functioned as a guide and judge throughout the annotation process. After the analysis of the relevant documents of the corpus (i.e., EHRs and tweets) by the medical experts, in conjunction with a review of comparable domain-specific schemata and guidelines such as COPD [19, 20], PhenoCHF [18] and i2b2 [43], the schema shown in Fig 2 was established by taking into account our chosen focus of annotating the complications associated with hypertension and diabetes. The medical doctor was asked to determine the entity types relevant to the task (explained in Table 2).

**Table 2:** Annotated entity classes in the PrevComp corpus.

Entity type	Description	Examples
Macrovascular	Complications that are caused by damage to the large- and medium-sized blood vessels (arteries and veins). Both diabetes and hypertension contribute to endothelial damage, which creates a basis and later accelerates the process of atherosclerosis and thickening of the arterial wall.	Coronary artery disease, peripheral arterial disease, and stroke
Microvascular	Complications that are caused by damage to the small blood vessels (arterioles, venules, and capillaries). Chronic hyperglycemia affects the endothelium of small blood vessels through several pathophysiological pathways.	Diabetic nephropathy, neuropathy, and retinopathy
Risk Factors	Factors that contribute to the progression of existing vascular disorders. They can be modifiable or nonmodifiable.	Modifiable: obesity, sedentary lifestyle, hypercholesterolemia, smoking, etc. Nonmodifiable: age, genetic predisposition (family history), ethnicity, and race.
Preventions	Lifestyle or social behaviors that put the patient at lower risk of developing the complications or progressing toward the macrovascular and microvascular medical conditions.	Weight loss, exercise, smoking cessation, avoiding stress, and adherence to therapy.

To ensure annotation quality and consistency, the development of the annotation guidelines went through an iterative process. The first draft of the annotation guidelines was written and shared with the annotators who were both medical doctors to independently annotate a random set of 20 EHRs and 50 tweets from the corpus. An analysis of the disagreements between the annotators in this annotation task was used to revise and update the guidelines. The most common source of disagreement between the two annotators was that one of the annotators annotated only the first occurrence of the entity mention rather than annotating all mentions of the entities. In addition, the annotators discussed whether to annotate the macrovascular and microvascular medical conditions when they were not developed as a result of complications of having hypertension and diabetes but were due to other reasons in the patient’s history, such as other diseases or genetic background. This disagreement was resolved by the medical expert who functioned as a judge to resolve any issues during the annotation process. The medical expert suggested that the macrovascular and microvascular mentions be annotated only if they developed as a consequence of the progression of hypertension and diabetes. Furthermore, the two annotators disagreed on the annotation of psychiatric disorders. One annotator thought that they negatively affected the status of any medical condition, while the other thought that they were not directly linked to the complications as risk factors. The medical expert advised annotating all the psychiatric disorders that increase disease progression as risk factors. In addition to the guidelines, regular meetings were conducted to discuss the guidelines and annotations and to answer any questions or concerns.

The revised guidelines were used to annotate the corpus. All the documents in PrevComp were annotated by two medical doctors for the entity types related to the complications of hypertension and diabetes by using the same set of annotation guidelines and schema. The annotation included marking up all entity mentions in the corpus related to the four semantic types mentioned in Table 1.

Following the manual annotation of entities, annotated entities in the corpus were mapped by the senior medical expert (who worked as a judge for the annotation projects) to semantic types in the Unified Medical Language System (UMLS) [44], a large-scale terminological resource of biomedical terminology that covers all entity types in our scheme, by using the MetaMap tool [45], as shown in Fig 2. For mapping the annotated terms to UMLS concepts, we followed the work reported on the following references [46, 47]. This step of mapping enriched our corpus with links to UMLS concepts and facilitated research on machine learning-based normalization methods to automatically integrate information on disease complications obtained from EHRs and Twitter.

### C. Annotation tool

LightTag (https://www.lighttag.io/) was used, as it was very easy to configure and user-friendly for our annotators, who were nontechnical users.

## IV. Results and discussion

To ensure that the generated corpus is of high quality, the annotations provided in the corpus should closely follow the guidelines set by the experts. The standard means to ensure the high quality of a corpus is to provide evidence regarding the reliability of the annotations in the corpus by calculating a statistic known as interannotator agreement (IAA). A high IAA score proves that the two annotators understood the task and provided consistent annotations when they worked independently. Furthermore, a high IAA score provides assurance that the corpus annotations are reliable and of high quality. The simplest way to calculate the IAA score is to calculate the absolute agreement by using the following formula [48]:

Number of agreed annotations / Total number of annotations.

However, this method of calculating an IAA is not accurate, as it does not take into account that a certain proportion of the agreement between the annotators occurred by chance. For the purposes of calculating IAA in this study, we followed a number of other related studies [49–51] by calculating IAA in terms of precision, recall and F-score. The F-score is the harmonic mean of precision and recall scores, which is normally calculated to compare the performance of an information retrieval or extraction system to a gold standard. The F-score is the same whichever set of annotations is used as the gold standard [51, 52]. To carry out such calculations, the set of annotations produced by one of the annotators was considered the ‘gold standard’, i.e., the set of correct annotations and the total number of correct entities was the total number of entities annotated by this annotator.

The precision (P) measure is the percentage of the correct positive annotated entities annotated by the second annotator in comparison to the annotation produced by the first annotator, which was assumed to be the gold standard. The precision is calculated as the ratio between the true positive (TP) entities and the total number of entities annotated by the second annotator (the sum of TPs and false positives (FPs)).

P=TP/TP+FP

Recall (R) is the percentage of positive annotated entities recognized by the second annotator. It is calculated as the ratio between the TP and the total number of annotations in the gold standard (the sum of TPs and false negatives (FNs)).

R=TP/TP+FN

The F-score is the harmonic mean of precision and recall and is calculated as follows:

F‐score=2*(Precision*Recall)/Precision+Recall

We calculated separate IAA scores for term annotation in the EHRs and tweets. Tables 3 and 4 report the IAA rates achieved in the PrevComp corpus, differentiating between agreement levels according to text type and between agreement rates for exact matching (i.e., the span of annotated terms must match exactly) and relaxed matching (i.e., the span of annotated terms needs only overlap with the corresponding term in the gold standard).

**Table 3:** IAA for EHRs using exact and relaxed matching criteria.

	Exact			Relaxed		
	P	R	F-score	P	R	F-score
**Macrovascular**	0.543	0.572	0.557	0.588	0.580	0.584
**Microvascular**	0.501	0.521	0.510	0.634	0.693	0.662
**Preventions**	0.618	0.582	0.601	0.694	0.591	0.638
**Risk Factors**	0.503	0.517	0.509	0.524	0.574	0.547
**Macro-average**	0.541	0.548	0.544	0.610	0.609	0.608

**Table 4:** IAA for tweets using exact and relaxed matching criteria.

	Exact			Relaxed		
	P	R	F-score	P	R	F-score
**Macrovascular**	0.566	0.906	0.697	0. 568	0.909	0.700
**Microvascular**	0.789	0.692	0.737	0.672	0.763	0.847
**Preventions**	0.745	0.847	0.793	0.860	0.761	0.808
**Risk Factors**	0.685	0.598	0.639	0. 612	0.697	0.652
**Macro-average**	0.696	0.761	0.717	0.678	0.783	0.752

The F-scores for exact matching were generally lower than those for relaxed matching due to disagreements between the annotators with regard to the exact span of the annotations. For example, most of the time complication terms are expressed within lengthy sequences of words and sometimes to complete a sentence, e.g., “cholesterol is significantly elevated” or “concentric left ventricular hypertrophy”. The annotators disagreed on the span or whether the modifiers should be included within the annotated text spans. For example, while one annotator marked “ischemia in the inferior wall” as an expression that corresponded to macrovascular complications of the interactions between hypertension and diabetes, the other annotator marked only "ischemia". As shown in Tables 3 and 4, the F-scores for relaxed matching were generally higher than those for exact matching due to disagreements between annotators with regard to the exact span annotated, which proves that the overall consistency between the two annotators was high.

The macrovascular and microvascular semantic types were the main source of inconsistency between the two annotators. This was mainly because of the broad definition of the macrovascular and microvascular complications which led to disagreement between the two annotators. For example, some of the diseases are related to the interaction between hypertension and diabetes but are not considered a direct consequence of the interactions, as hypertension may have many other causes depending on the patient case and medical history. These diseases that can be a consequence of the long-term interaction between hypertension and diabetes but also be caused by genetic disorders include congestive heart failure (CHF), congenital heart malformations, and pulmonary disorders. In those cases, the annotators disagreed such that the first annotator (i.e., who produced the gold standard set) did not annotate the terms if the cause was genetics or related to family history and only annotated the macrovascular terms if they happened as a consequence of the interactions between hypertension and diabetes. However, the second annotator annotated these terms as macrovascular and microvascular complications. Another example of disagreement and the main cause of low F-scores occurred due to repetitive occurrences of the complications, which caused the annotator to occasionally miss annotating some of the relevant terms.

The reason for the low F-scores for the risk factor was because the annotators sometimes disagreed on the risk factors. For example, one of the annotators annotated alcohol use as a risk factor, whereas the other annotator felt that using alcohol was not necessarily a risk factor unless the patient frequently abused alcohol, which would be noted in the corpus as reflecting lifestyle information of the patient.

It was noticed that the agreement with the EHRs was lower than the agreement with the tweets, which was due to the short length of the tweets (i.e., 280 characters per tweet), making it very easy for the annotators to read through the tweets very quickly and mark up all the mentions that were relevant to the task. In contrast, the annotation of the EHRs was more complex and required extra effort: 1) the topic specificity required the annotator to read and analyze the patient information and then decide whether the medical conditions were considered consequences and complications related to hypertension and diabetes; 2) the text in the EHRs was longer than the text in the tweets and more complex, as it included more than one section, such as medical history, laboratory data, physical exams, medications, and other information, and the annotator sometimes needed to read the full text of the report more than once to correctly decide and annotate the relevant information.

PhenoCHF [18] and COPD [19] shared the following characteristics with PrevComp:

- Both the PhenoCHF and COPD corpora consist of heterogeneous text (i.e., EHRs and full scientific articles from the literature).
- Pure manual annotation was used as an approach to annotate PhenoCHF, and manual annotation was partially used to annotate COPD.
- PhenoCHF and COPD were annotated for phenotypic information, and we noticed that both phenotypic concepts and chronic disease complications were mentioned in full phrases, e.g., a decrease in the rate of lung function and increased shortness of breath.

In comparison to the related annotation effort results regarding PhenoCHF and COPD [46, 53], the results of our annotation were satisfactory considering the complex nature of the task where the annotators were unable to rely only on the mentions of the medical conditions. They needed to fully read and analyze the information to decide whether the mentioned medical condition(s) was a complication of chronic disease.

## V. Conclusion

This paper presents a detailed description of our procedure for the development of the PrevComp corpus, including the annotation schema and guidelines. The corpus consists of 274 EHRs and 2,265 tweets and is novel in its domain-specific topic, which is related to the complications of two of the most common chronic diseases, as well as the prevention strategies and risk factors that could contribute to decreasing the incidence of complications. The corpus is also unique in its integration of two different text genres and document types (EHRs and tweets). The generated corpus can serve as a gold standard for the development of TM tools that can extract and integrate important information from both text types. For example, the PrevComp corpus can be used to develop named-entity recognition (NER) techniques on a large scale to extract disease complication information from both EHRs and Twitter. Additionally, it can be used to develop novel methods to normalize disease complication concept mentions from heterogeneous textual sources (i.e., EHRs and Twitter) and map them to UMLS concepts.

## Supporting information

S1 File Annotation guidelines. (PDF)

S2 File (ZIP)
