# Supplementary material for: Building a semantically annotated corpus for chronic disease complications using two document types
Source: PLoS One. 2021 Mar 18;16(3):e0247319. doi: 10.1371/journal.pone.0247319 (PMC7971867; doi:10.1371/journal.pone.0247319)
Supplement: S1 File — (PDF) [file pone.0247319.s001.pdf]

Annotation Guidelines

Diabetes and hypertension are strongly associated with both microvascular and macrovascular complications, including retinopathy, nephropathy, and neuropathy (microvascular) and ischemic heart disease, peripheral vascular disease, and cerebrovascular disease (macrovascular), resulting in organ and tissue damage in approximately one third to one half of people with diabetes.

The goal of this annotation task is to go through the corpus which consists of two document types (tweets and EHRs) related to the complications of hypertension and diabetes and annotate the mentions related to the complications of hypertension and diabetes, risk factors that put the patients at higher risk to develop the complications as well as prevention measures that could contribute to lower the possibility of the development of the complications or prevent it.

Annotation task

Annotation is marking up piece of text with tag to describe it. The aim of this task is to annotate the following terms:

1- complications of hypertension and DM which includes marking-up all the mentions of both macrovascular and microvascular diseases.

- Example of macrovascular:

he is a very pleasant 60 year old female with cardiovascular risk factors of diabetes mellitus, hypertension, peripheral arterial disease , stroke , coronary artery disease

- Example of microvascular :

The proteinuria, if confirmed, would support the diagnosis of diabetic nephropathy

2- Risk factors which is medical or social conditions that put the patient at higher risk to develop the complications such as bad life style (i.e. physical inactivity, smoking, being overweight).

Examples:

obesity, sedentary lifestyle, hypercholesterolemia, smoking, age, family history of chronic diseases etc.

- she is obese.
- ack year cigarette smoking history
- the patient is old lady.

3- Prevention which includes any practice that could slow down the progress of the complication and put the patient at lower risk to develop or worsen the condition of the complications.

Examples:

Weight loss, exercise, smoking cessation, avoiding stress, adherence to therapy.

- the patient follows regular exercise program
- She Follows Weight Watchers diet

General guidelines

- Only annotate the correct span (as much information as required).
- The complication mentions , risk factor and prevention term could be expressed in any syntactic structure it can be:
- **Noun phrase**

Hyperlipidemia

- **Prepositional phrase**

Increased in weight

Shortness of breath

- **Adjective phrase**

Myocardial infarction

Coronary artery disease

Annotate all abbreviations and acronyms that refer to the required annotation in the task as mentioned above. For example: CHF, CAD , CKD, MI

If the medical condition is preceded by modifier of multi words phrase.

- Annotate the whole phrase for example: Increased potassium.
- increased shortness of breath
- left atrial enlargement

<u>Do Not annotate the following:</u>

- Normal condition for example if the information describe normal function i.e. EKG showed normal sinus rhythm , regular chest etc
- anything you infer from the text; only annotate the explicitly mentioned entity. For example consider the following lab result:

Laboratory data: INR of 1.6, BUN of 110, creatinine 3, potassium 5.5, white blood cell count of 11.7 and a hematocrit of 27.9.

Do not annotate anything from the previous lab results.
